# Understanding innovation: The development and scaling of orange-fleshed sweetpotato in major African food systems

**DOI:** 10.1016/j.agsy.2019.102770

**Published:** 2020-03

**Authors:** Jan W. Low, Graham Thiele

**Affiliations:** a International Potato Center, Box 25171, Nairobi 00603, Kenya; b International Potato Center, CGIAR Research Program on Roots, Tubers and Bananas, C. Postal 1558, Lima 12, Peru

**Keywords:** Orange-fleshed sweetpotato, Innovation, Scaling, Biofortification, Sub-Saharan Africa, Micronutrient malnutrition

## Abstract

The development and scaling of orange-fleshed sweetpotato (OFSP) during the past 25 years is a case study of a disruptive innovation to address a pressing need – the high levels of vitamin A deficiency among children under five years of age in sub-Saharan Africa. When the innovation was introduced consumers strongly preferred white or yellow-fleshed sweetpotato, so it was necessary to create a demand to respond to that need. This was at odds with the breeding strategy of responding to consumers’ demands. Additional elements of the innovation package include seed systems and nutrition education to create the awareness amongst consumers of the significant health benefits of OFSP. Complementary innovation is required in promotion and advocacy to ensure a supportive institutional environment.

Four dimensions-- technical, organizational, leadership, and institutional environment-- are explored across five distinct phases of the innovation process, from the emergence of the innovative idea (1991–1996) through scaling phase in 15 countries under a major institutional innovation (2015-mid-2019), the Sweetpotato for Profit and Health Initiative (SPHI).

Systematically gathering evidence of nutritional impact and ability to scale cost-effectively was requisite for obtaining support for further development and diffusion of the crop. Positive findings from a major study coincided with a major change in the institutional environment which placed agriculture and nutrition at the forefront of the development agenda, resulting in an inflection point in both research and diffusion investment. The role of committed leadership during all phases was critical for success, but particularly during the first decade of limited support in a challenging institutional environment. The most critical technical achievement underpinning scaling was moving from 2 to 13 African countries having local breeding programs. Evidence is presented that adapted, well performing varieties which consumers prefer is the foundation for successful scaling to occur. Building a cadre of within country and regional advocates was critical for getting sustained commitment and local buy-in to the concept of biofortification by regional bodies and governments, which in turn built within country ownership and the willingness of donors to invest. The SPHI united diverse organizations under a common vision with a simple metric--- the number of households reached with improved varieties of sweetpotato. Since 2009, 6.2 million households were reached by July 2019 in 15 SSA countries. Much more remains to be done. Advocacy efforts led to the integration of nutritious foods into many national and regional policies, setting the stage for further investment.

## Highlights

• Vitamin A-rich, orange-fleshed sweetpotato is the lead biofortified crop in Africa.
• OFSP was a disruptive innovation as non-OFSP types were demanded before.
• Committed leadership for innovation for over 20 years was critical for scaling.
• A strong evidence base was requisite for obtaining donor support for scaling.
• Partner initiative for scaling united diverse organizations with a common vision.

## 1 Introduction

Orange-fleshed sweetpotato (OFSP) is an extremely rich plant-based source of pro-vitamin A (beta-carotene). Just 125 gms of a cooked OFSP root meet the daily vitamin A needs of a young child (Low et al., 2009). In sub-Saharan Africa (SSA), the dominant varieties preferred by consumers are white-fleshed, completely lacking in beta-carotene. The development and scaling of OFSP during the past 25 years is a case study of a disruptive innovation^1^ (Bower and Christensen, 1995) to address a pressing need – the high levels of vitamin A deficiency among children under five years of age, with a prevalence rate still around 48% in 2013 (Stevens et al., 2015). The challenge of addressing micronutrient deficiencies, or so-called *hidden hunger* is that it is not obvious to those who suffer from it. Hunger pangs are clearly associated with insufficient energy intake. But no one wakes up saying “I crave vitamin A today”. Thus, the traditional “demand-driven” strategies do not hold in this context; the model instead is one of demand creation. Because sweetpotato is a field crop in tropical SSA, OFSP became a model crop for the concept of *biofortification*, that is breeding for micronutrient enhancement of staple foods (Bouis, 2002). Biofortified crops are sources of energy as well as at least one key micronutrient. Since poor SSA households obtain 60–70% of their calories from staple foods, biofortified crops are excellent vehicles for combatting hunger and micronutrient deficiencies concurrently (Bouis, 2002).

Studies of adoption in agriculture historically relied on the diffusion of innovations theory, which describes how and at what rate new technologies spread (Rogers, 2003). Subsequently innovation science has given more attention to the networked nature of scaling and the institutional dimension or rules of the game which can drive innovation (Hall et al., 2003). The scaling literature distinguishes different periods in the innovation process (1) problem identification (2) proof of concept with evidence generated to convince stakeholders that the innovation is worthy of investment, (3) piloting of the innovation and (4) scaling, with emphasis on dissemination of the innovation to specific target groups (IFAD, 2015). Yet often projects fail to make it beyond piloting. Woltering et al. (2019) point out that “most pilot projects do not scale up to achieve wider impact, cease to exist after a (subsidized) demonstration phase, and fade out after initial funding ends”. Recently, in recognition that many agricultural technologies “stay on the shelf”, there is a greater effort to understand a given innovation’s “readiness-to-scale” and how to assure that scaling occurs (World Health Organization, 2010; Sartas et al., 2017). Moreover, the international donor community has been pushing for scaling to occur faster as a means of justifying their investment. For example, the US government's food security strategy calls for potential scaling pathways to be identified early in the research process and for enhanced collaboration with potential scaling partners as innovations advance through the adaptive research stages (Feed the Future, 2017).

Sweetpotato is a vegetatively propagated crop which is primarily grown for food security rather than sale in SSA^2^, and widely considered to be under the domain of women producers in East and Southern Africa. Vegetative propagation means it is easy for farmers to share and retain planting material without losing varietal characteristics. These features make it less appealing to private sector enterprises which could recover the cost of their investment into varietal promotion through seed sales. The opposite is true of the development and the diffusion of hybrid maize a cash crop in the USA, *the* classic example described in Rogers (2003). Hybrid maize was developed by public sector research universities, but they were a boon to private sector seed companies, as to maintain their yields farmers must purchase new seed every year. Even with consistent extension service and private sector seed, fertilizer, and pesticide company support, it took 13 years to get widespread adoption of the technology once it was available.

The only major study comparing adoption among 20 crops of improved varieties bred by CGIAR centers presents data as of 2010 (Walker and Alwang, 2015). Highest varietal uptake was seen among the cereal crops. Among the vegetatively propagated crops, only cassava was above the area-weighted grand mean adoption level of 35% for improved varieties. Sweetpotato’s adoption level was just 6.9% for improved varieties, increasing to 24% if local landrace releases were included. The spread of improved cassava materials was attributed to substantial public sector invested in dissemination. The author’s found that varietal turnover on average ranged from 14–25 years. This and the hybrid maize experience suggests that the time frame required for scaling of an innovation such as OFSP might be quite significant.

Much innovation research focuses on the steps that the adopter of the technology goes through, namely knowledge, persuasion, decision, implementation, confirmation, but documentation of how innovations came into being, under a range of agro-ecological, cultural and socio-economic settings has been less studied. Understanding the development and promotion of OFSP in SSA as an innovation which has achieved broad uptake beyond the original pilots is a unique opportunity to study the scaling of an integrated agricultural-nutrition intervention. In this case, demand creation for behavioral change and engagement at the policy level and with investors was just as critical to driving uptake as the technological innovations to improve agronomic performance and consumer acceptability of the improved varieties.

## 2 Method

Five time periods can be distinguished in the development and scaling of the OFSP innovation package: (1) the emergence of the innovative idea (1991–1996); (2) proving the potential of the innovation to the nutrition community (1997–2005); (3) evaluation of the potential to scale cost-effectively (2006–2009); (4) significant investment in research to address breeding and other bottlenecks initiated and launching of Sweetpotato for Profit and Health Initiative (SPHI) (2010–2014) and (5) expanded dissemination at scale (2015-mid-2019). The understanding of the innovation process during this period draws heavily on key informant knowledge, particularly from the lead author of this paper and others involved in the development and diffusion of OFSP, published literature, and project reports and briefs. Key sources are presented in Table 1.

**Table 1:** Sources of information for different phases of the innovation process.^a^

Time period	Published references	Other sources
Entire period	Low et al., 2007; Low et al. (2007b)	▪ Key informant knowledge (lead author); CIP and HarvestPlus scientists involved in biofortified crop development; Anna Herforth (Ag2Nut web-based Working Group)
Phase 1. 1991–1996 Emergence of the Innovative Idea	Low et al. (1997); Hagenimana *et al.* (1999a); Hagenimana *et al.* (1999b)	▪ CIP annual report (1998)
Phase 2. 1997–2005 Proving the Potential to the Nutrition Community	Jalal et al. (1998); Bouis (2002); Graham et al. (2001); Haskell*et al.* (2004); van Jaarsveld et al. (2005); Low et al., 2007; (Low et al., 2007b); Low and van Jaarsveld (2008)	▪ Donor report: Low *et al.* (2006) ▪ Low *et al.* (2001) ▪ Working paper: CIP (2007) Five Years of Vitamin A for Africa
Phase 3. 2006–2009 Potential to scale cost-effectively	Andrade et al. (2009); Hotz *et al.* (2012a); Hotz *et al.* (2012b); Jones and de Brauw (2015)	▪ Donor report: Arimond et al. (2010); ▪ Donor report: Low *et al.* (2010)
Phase 4. 2010–2014 Significant Research Investment; SPHI Initiated	Low (2011); Girard et al. (2012); Ruel et al. (2013); Low et al. (2015); Laurie et al. (2015); Webb-Girard et al. (2017); Grüneberg et al. (2015); McNiven et al. (2016)	▪ Annual SPHI Briefs, available on www.sweetpotatoknowledge.org (SKP) ▪ CIP-led project reports ▪ Donor report: CIP (2015) Mama SASHA
Phase 5. 2015-mid-2019 Scaling under the SPHI	Ministério da Agricultura e Segurança Alimentar (Mozambique) (2015); de Brauw et al. (2018); Covic et al. (2017); Bouis et al. (2017); Ruel et al. (2017)	▪ Annual Status of Sweetpotato in SSA report (On SPK) ▪ Annual SPHI Briefs (On SPK) ▪ Minutes of SPHI Steering Committee ▪ CIP-led project reports ▪ Minutes and presentations from 4 Technical community of practice working groups (On SPK)

a For the complete citation on the articles and donor reports, refer to the references.

In examining the scaling of the OFSP innovation package, four critical dimensions are explored. The **technical** dimension examines how innovation design evolves over time to respond to new information and different needs of end-users in various locations. The **organizational** dimension considers how the kind and numbers of organizations involved change as the innovation goes to scale, the scaling partners (e.g. government, private, development) involved and how the process was organized. The **institutional environment** dimension looks at the policies and strategies or changes that facilitated investment in the innovation, enabling affordable access to a significant number of end users. Finally, the **leadership** dimension, recognizes that champions of innovations play a key role in their development and uptake. In the business world, Gliddon (2006) established the concept of understanding and measuring the competencies underlying innovation leadership. Private sector companies have increasingly recognized that valuing and promoting innovative thinking and leadership styles that can successfully evaluate a generated idea then move it into implementation is key for continued organizational performance. After reviewing key features of each dimension over time, the following questions will be addressed:

1. What is the role of “leadership” in driving an innovation process?
2. What is the role of evidence in scaling?
3. What are the critical inflexion points in a long-time scale innovation process?
4. What is the role of the public sector in scaling?
5. What are the key elements of the institutional environment in supporting scaling?
6. What new research needs appear and are addressed as scaling happens?

In addressing the concept of scaling, we use Hartmann and Linn’s definition: “Scaling up means expanding, adapting and sustaining successful policies, programs and projects in different places and over time to reach a greater number of people” (Hartmann and Linn, 2008). There is no agreement as to what the cut-off point is for determining what number of people constitutes “scale” and in the case of OFSP, there is considerable variation between countries due to need for localized adaption of the main technology, the OFSP varieties themselves.

## 3 Results

This section highlights the key factors occurring during each phase of the OFSP innovation story to date. The technical dimension is the most complex and the various components are presented in further detail in Table 2. The remaining three dimensions are described by phase in Table 3, which also includes the number of beneficiary households reached, i.e. that received planting material or “seed” of improved sweetpotato varieties, an indicator that has proved to be the one that can be collected across a diverse set of organizations, using different delivery models. It is not a measure of adoption.

**Table 2:** Summary of progress by period of technical dimension of the innovation package.

Time periods	Phase 1. 1991–1996 Emergence of the Innovative Idea	Phase 2. 1997–2005 Proving the Potential to the Nutrition Community	Phase 3. 2006–2009 Potential to scale cost-effectively	Phase 4. 2010–2014 Significant Research Investment; SPHI Initiated	Phase 5. 2015–2018 Scaling under the SPHI; Continued Research Investment
Technical Components of the Innovation Package					
1) Varieties with high beta- carotene adapted to local growing conditions and consumer preferences PoC: Proof-of-Concept	***Recognition of potential for OFSP to be source of vitamin A for Africa***. PoC with introduced varieties from outside Africa & local orange landrace in Kenya; Networks for varietal selection from best bets in East & Central (since 1991) and Southern Africa (since 1994)	Advanced clones bred in Peru collapse when introduced into SSA in 2002. ***By 2004-2005, recognition of need to breed in Africa for Africa***	6 new OFSP varieties bred in Africa released by 4 SSA countries. PoC: Accelerated Breeding Scheme (ABS) begins in Peru and Mozambique	1. 1.40 new OFSP varieties bred in Africa released by 9 SSA countries 2. 3 population development platforms launched by CIP: Uganda for virus resistance, Mozambique for drought tolerance & Ghana for low-sweet sweetpotato 3. Initial scaling: 10 countries using ABS	12 new OFSP varieties bred in Africa released by 5 SSA countries PoC-2019: bioavailability study for high iron OFSP PoC: 2010-2019 Evidence that hybrid breeding approaches can be applied to sweetpotato with significant genetic gains Use of ABS standard practice
2) Pre-basic seed provision	**Existing practice**: 12-24 months to remove viruses in thermotherapy; tissue culture multiplication; followed by hardening and screenhouse multiplication. Many countries without pathogen tested pre-basic seed.	***Recognition of significant delays to remove viruses and get clearance to move materials from Peru to Africa.***	Greater CIP collaboration with Plant Quarantine center at KEPHIS in Kenya as a regional hub for virus removal from sweetpotato varieties and serve Africa for distribution of disease-free starter material.	***Recognition of need to speed up pre-basic seed production & use business model approach for public sector entities*** Iso-accreditation of regional germplasm center at KEPHIS. Development of better diagnostic tools for virus detection initiated.	11 NARES cost out production costs, establish rotation funds & develop business plans for sustainable pre-basic seed production. Two better diagnostic tools available at end of 2018. ***Recognition that better linkages between basic multipliers and pre-basic seed providers needed.***
3) Access to seed at the community level	Group level vine multiplication ***Recognition of problem of degeneration of planting material due to virus especially in bimodal areas.***	Development efforts: *community-level decentralized vine multipliers (DVMs);* Emergency projects: *Mass dissemination from centralized site*	***Recognition of challenge of retaining seed during the dry season:*** PoC: Triple S research Pilot of DVM and Mass dissemination models at scale under REU	Validation of Triple S PoC: Net tunnels to prevent virus infection of basic starter seed of multiplier in high virus pressure areas.	Initial Scaling of Triple S Comparative study of DVMs in Tanzania & Uganda; extended to 6 other countries. Validation & initial scaling of net tunnels; challenges with sustained management; Mini-screenhouses shown to be superior to net tunnels.
4) Integration with community level nutrition education (NE)	PoC: community-level nutrition education essential for increasing frequency of intake of vitamin A rich foods in young child diet	PoC: Integrated ag-nutrition marketing model tested in central Mozambique, showing impact on vitamin A intakes and status in young children	Validation of one year of group level NE as sufficient for OFSP integration into young child diet. Initial scaling to 24,000 in Uganda & Mozambique using integrated ag-nutrition model cost-effective	PoC: NE is integrated into ante-natal care counseling for pregnant women at health facilities, plus continued group sessions. Impact on vitamin A intakes with partial participation; Impact on stunting and vitamin A status with full participation.	Initial scaling of integrated ag-nutrition-health model. PoC effort in Ethiopia, adds “Healthy tool kit” into NE program to provide more guidance as to amounts to be fed to children <2 yrs old at different stages and as to porridge thickness.
5) Product promotion & placement	Not significant	Initiation of orange brand and slogan (Mozambique); promotion materials	Use of orange branding expanded to other countries; Better understanding of willingness-to-pay	Advocacy toolkit and annual briefs of progress in research & dissemination; presence at global events	Greater use of social media and continued promotion at country level, regional and global events
6) Post-harvest innovations for diversified use	Incorporation of OFSP purée into chapatis/donuts	PoC on OFSP puree for partial wheat flour substitution in bread in Mozambique	Piloting of OFSP products in several countries	Validation of OFSP puree as partial wheat flour substitute with commercial enterprise	Initial scaling of OFSP puree for different baked products in several countries; shelf-storable puree developed
7) Expanded delivery mechanisms		OFSP with one-shot nutrition campaign as part of emergency response to floods and drought		PoCs: Integrated agriculture-nutrition-health & market driven value chain for OFSP processed products	PoC: OFSP integrated into school feeding program in Nigeria and schoolbooks linked to vine access in Uganda

**Table 3:** Summary of progress by period of the non-technical dimensions and number of beneficiary households reached.

Time	Organizational	Institutional Environment	Leadership	Number of Countries & Beneficiary Households Reached
Phase 1. 1991–1996 Emergence of the Innovative Idea	1. International Potato Center (CIP) SSA Program 2. Limited support to NARS through networks 3. Bouis initiative: CGIAR Micronutrients Project began in 1994	1. Period of disinvestment in agriculture 2. Growing interest in combatting micronutrient deficiencies, with heavy emphasis on supplements	Low: CIP post-doc recognizes OFSP potential for SSA Mwanga: National program breeder in Uganda Maria Andrade (Andrade) obtains doctorate in sweetpotato breeding from North Carolina State University (NCSU) in 1994	Best bet OFSP materials introduced into 18 countries via networks < 5000 households
Phase 2. 1997-2005 Proving the Potential to the Nutrition Community	1. CIP launched Vitamin A for Africa Partnership (2001-2006) 2. HarvestPlus- Biofortification Challenge Program Phase 1 began in 2003	1. In 1999, Mozambique approved its first strategy for combatting micronutrient deficiency. 2. Lowest period of public agriculture investment in modern history 3. Nutrition community calls for more evidence for food-based approaches; strong support to capsule supplementation continues	Andrade working with IITA in Mozambique in 1996. Meets Low, who began policy work for International Food Policy Research Institute in Mozambique in mid-1996. Mwanga obtains doctorate from NCSU in 2001. Wolfgang Grűneberg joins CIP as leader of global sweetpotato breeding in 2004. Develops accelerated breeding scheme idea in 2005. Low, while working for Michigan State University in Mozambique, obtains funding for OFSP proof-of-concept study for impact on vitamin A intakes & status. Leads study 2002-2005. Low returns to CIP as Regional Director for SSA in late 2005.	Mozambique: >1 million Uganda: <10,000 Other SSA <3,000 Opportunity for dissemination opens in Mozambique as response to major floods in 2000-2001
Phase 3. 2006-2009 Potential to scale cost-effectively	1. HarvestPlus-led Reaching Endusers proof-of concept scaling study; CIP sub-grantee 2. HarvestPlus began Phase 2 in 2009	1. 2008 food price crisis re-ignites interest in agriculture sector 2. 2008 Lancet series on maternal and child undernutrition makes a convincing case for nutrition investment	1^st^ OFSP varieties bred in Ugandan released in 2007 (Mwanga) Andrade joins CIP as breeder for Southern Africa in 2006 Mwanga joins CIP as breeder for East & Central Africa in 2009 Low leads Sweetpotato Action for Security and Health in Africa (SASHA) proposal development and SPHI design	24,000 household beneficiaries in Mozambique & Uganda By the end of this period, dissemination activities initiated in 9 countries
Phase 4. 2010-2014 Significant Research Investment; SPHI Initiated	1. CIP received funding for SASHA Phase 1 in mid-2009 2. CIP launched Sweetpotato for Profit & Health Initiative (SPHI). Executive Steering Committee oversaw SASHA and SPHI in October 2009 3. HarvestPlus began Phase 3 (2014-2018) 4. CIP received grant (Reaching Agents of Change) from Bill & Melinda Gates Foundation to invest in training advocates and capacity strengthening (2011-2013)	1. 2010 launch of the SUN movement, where David Nabarro coined the terms "nutrition-sensitive" and "nutrition-specific". The concept of "nutrition-sensitive" as a springboard for work within *many* organizations to justify an increased focus on nutrition in agriculture. 2. Ag2Nut discussion group & key recommendations document in 2010 3. 2012 UK Sponsored Global Hunger Event linked to Olympics 4. 2013 Lancet series: calls to combat malnutrition in all of its forms & use multi-sector approaches 5. CAADP process integrates nutrition (2011-2013)	1^st^ OFSP varieties bred in Mozambique using ABS released in 2011 (Andrade); Low manages SASHA Phase 1 and leads SPHI Phase 1 Commitment to building strong sweetpotato community of practice in SSA (Low, Andrade, Mwanga & other members of SASHA team)	1.13 million households reached in 14 out of 17 SPHI target countries in SSA. (Kenya, Uganda, Tanzania, Rwanda, Ethiopia, Mozambique, Malawi, Zambia, South Africa, Nigeria, Burkina Faso, Ghana, Angola, Madagascar) Direct beneficiaries defined as receiving vines of improved varieties through a project; indirect through farmer-to-farmer or markets
Phase 5. 2015-mid-2019 Scaling under the SPHI	1. SASHA Phase 2 (2014-2019) supported by SASHA Project Advisory Committee 2. 2^nd^ Phase of SPHI, co-lead by CIP and Forum for Agricultural Research in Africa (FARA), supported by SPHI Steering Committee 3. By 2019, 6 research organizations; 6 implementation organizations and one private-sector company on SPHI Steering Committee	1. UN Decade of Action on Nutrition (2016-2025) 2. Sustainable Development Goals by 2030 for ending hunger & all forms of malnutrition 3. EAT Lancet study (2019) calling for massive reform of food systems 4.2011-2019, biofortification/nutritious foods integrated into policies: 7 regional; 23 national agriculture and18 national nutrition	Low, Andrade, Mwanga along with Bouis win 2016 World Food Prize for Biofortification Low manages SASHA Phase 2 and co-leads SPHI Phase 2 2^nd^ batch OFSP varieties bred in Mozambique using ABS released in 2016 (Andrade).	Additional 4.18 million households reached in 12 countries. 5.3 million since 2009; 6.2 million by July 2019. Spillover with other organizations into non-target countries in Ivory Coast, Senegal, Sierra Leone, and Somalia

### 3.1 Phase 1: The Emergence of the Innovative Idea (1991–1996)

#### 3.1.1 Institutional environment dimension

The 1990s was a time of declining resources for agriculture in SSA, both domestic and from overseas development assistance (ODA). Furthermore, many African governments did not prioritize sweetpotato, a crop mostly grown by women in poorer rural households; instead governments and donors prioritized cereal crops, particularly maize.

In contrast, by the early 1990s research findings showing how supplementation with vitamin A capsules led to 20–30% reductions in child mortality had energized the nutrition community to tackle vitamin A deficiency as a key priority. Prevalence of vitamin A deficiency among children under five years of age was estimated to be 44.4% in Africa, affecting 56.4 million children (World Health Organization (WHO), 2009). At the 1993 International Vitamin A Consultative Group (IVACG) meeting, a policy statement was issued highlighting the importance of vitamin A status for child survival (Reddy, 2002). Vitamin A capsule supplementation, ideally twice a year, began to be integrated as key component in child survival programs. Moreover, IVACG meetings in the early 1990s concluded that low consumption of Vitamin A rich vegetable and fruits was driven by low availability and low awareness of their value at the household level. This led to funding of several gardening and communication studies and triggered research to determine carotenoid bioavailability in specific foods and improve carotenoid retention (Reddy, 2002).

#### 3.1.2 Organizational dimension

At this time, there existed 15 international agricultural centers belonging to the Consultative Group for International Agriculture Research (CGIAR). Each center had their specific mandates. The International Potato Center, known by its Spanish acronym CIP, had the global mandate for potato, sweetpotato, and Andean root and tuber crops, with its headquarters in Peru. Varietal testing and other research activities in sub-Saharan Africa (SSA) were principally carried out by national program partners linked to USAID-financed networks.

In Africa during this time, there were only two countries truly breeding sweetpotato (i.e. making parental crossings to generate new clones)—South Africa and Uganda (the top sweetpotato producing country in SSA at that time). The breeding work in South Africa focused on frost tolerance and traits desired for commercialization whereas in Uganda^3^ it focused on virus and weevil resistance for white or yellow-fleshed varieties.

#### 3.1.3 Technical dimension

All breeding (making actual crosses) conducted by CIP was at its headquarters. Promising varieties or clones were sent to the regions for selection of the “best bets”. Sweetpotato breeding focused on selection for yield, early maturing, and virus resistance. In SSA, CIP provided a range of “best-bet” varieties of all flesh colors for national programs within these networks to test.

The high beta-carotene content in just 100 g of orange-fleshed varieties made OFSP a potential candidate for addressing vitamin A deficiency (Low, 2013). The key technological challenge which initially emerged was that in the extant orange-fleshed germplasm introduced from outside Africa, there was a strong negative correlation between beta-carotene content and dry matter content. The darker the orange color, the greater the beta-carotene content; but the introduced darker orange varieties had low dry matter content (18–22%). Adult consumers in East and Southern Africa found these types of OFSP varieties to be watery, preferring high dry matter content (27–30+%), even though many were higher yielding than the local varieties.

#### 3.1.4 Leadership dimension

During this phase, four scientists emerged on the scene who were to become the drivers underlying the promotion of orange-fleshed sweetpotato as a biofortified crop. Two were agricultural economists, Jan Low and Howarth Bouis, and two were sweetpotato breeders, Robert Mwanga and Maria Andrade. Understanding their roles and the importance of the multi-disciplinary nature of the development of the OFSP innovation is critical to this case study.

Low, the lead author of this paper, joined CIP’s regional office in Nairobi in a post-doctoral position in 1994. Having minored in nutrition during her doctoral studies, Low recognized the high potential for making a major public health impact on vitamin A deficiency through the introduction and promotion of orange-fleshed sweetpotato types. However, at the time, CIP engaged in “demand-led” breeding, which meant focusing on red-skinned, white-fleshed varieties dominant in East Africa.

In 1995, Low obtained a small grant ($75,000) to collaborate with the Kenya Agriculture Research Institute on a two-year study to compare OFSP uptake among ten women’s groups who received two orange-fleshed varieties and one yellow-fleshed variety and agricultural extension advice *only* to ten women’s groups who received the same agriculture package plus nutrition education (Low et al., 1997). A key outcome indicator was the frequency of consumption of vitamin A rich foods (a semi-quantitative index) (Rosen et al., 1994). Results indicated that the nutrition education component was essential for seeing an increase in this index among children under five years of age (Hagenimana et al., 1999), a finding that would influence the design of future interventions. Moreover, upon analysis, the yellow-fleshed variety had no significant amounts of beta-carotene. Henceforth, only orange-fleshed varieties would be employed for vitamin A efforts by CIP.

The orange color was liked by all household members, but children preferred the easier to swallow, low dry matter varieties, while adults preferred those with higher dry matter. The fact that women dominate smallholder sweetpotato production in East and Southern Africa and also are the principal caregivers in their households increased the potential for a successful integrated agriculture-nutrition intervention.

During the same period, Bouis, based at another CGIAR center, the International Food Policy Research Institute (IFPRI), began exploring the idea of developing and releasing high vitamin and mineral varieties of staple crops. Many breeders within the crop CGIAR centers felt there would be significant trade-offs between having high vitamin and mineral contents and high yields. Bouis held a USAID-funded workshop in 1994 to look for CGIAR scientists and donors to support such an effort. Breeders at the CGIAR centers of IRRI (rice), CIAT (beans and cassava), and CIMMYT (maize and wheat) expressed interest and limited funding was obtained to explore the feasibility of enhancing vitamin and mineral content using conventional breeding (HarvestPlus, 2018).

Mwanga was the lead sweetpotato breeder at the National Crops Resources Research Institute (NaCRRI) in Uganda. The breeding program focused on selecting and breeding for virus and weevil resistance. In 1995, Uganda released five local landraces and one cream-fleshed improved variety. Mwanga was supported by the McKnight Foundation to pursue a doctoral degree in sweetpotato breeding at North Carolina State University (USA) from 1995 through 2002.

Andrade also did her doctoral degree in sweetpotato breeding at North Carolina State University (1989–1994), and in 1996 was employed by the International Institute of Tropical Agriculture (IITA) to coordinate the Southern Africa Root Crops Research Network for cassava and sweetpotato in Southern Africa, based in Mozambique.

### 3.2 Phase 2: Proving the Innovation’s Potential to the Nutrition Community (1997–2005)

#### 3.2.1 Institutional environment dimension

At the IVACG 1999 meeting in Durban, South Africa, the major donors were clearly aligned to support capsule supplementation to combat vitamin A and iron micronutrient deficiencies at scale. Food-based approaches were criticized for having insufficient evidence of impact on nutritional status. However, the debate concerning bioavailability of carotenoids subsided with the publishing of *The Bioavailability of Dietary Carotenoids: Current Concepts (*International Vitamin A Consultative Group (IVACG), 1999*;*Reddy, 2002*).* New standards for converting carotenoids to retinol (vitamin A) were provided and research on developing conversion rates for individual foods continued, with greater attention paid to the food matrix in which they were consumed. Recognizing that nutrition continued to be underfunded at the start of the 21^st^ century, actors within the nutrition community began a more concerted effort to reposition nutrition as central to development.

Concurrently, policy makers were beginning to recognize that more needed to be done to get the agriculture sector moving in SSA (The World Bank, 2007) and key African leaders recognized the need for increased public investment and commitment to agriculture. This led to the launching of the Comprehensive Africa Agriculture Development Programme (CAADP), a joint initiative of the African Union and the New Partnership for Africa’s Development (NEPAD) in 2003. CAADP set specific goals for African governments to commit to allocating 10% of their national budgets to agriculture (Kimenyi et al., 2013).

However, there was still limited awareness of the potential contribution of agriculture to nutrition. With the publishing of a major paper in *Advances in Agronomy* (Graham et al., 2001), providing evidence that staple food micronutrient quality could be enhanced through breeding or agronomic practice (i.e. fertilizers), more breeders and donors began to be convinced about the viability of biofortification^4^. It is of note, however, that OFSP was not mentioned in this paper.

#### 3.2.2 Leadership dimension

Low joined the International Food Policy Research Institute (IFPRI) in late 1996 as a research fellow based in Mozambique and was able to link the government’s nutrition unit to Andrade’s OFSP varietal selection work, obtaining support in 1997 for the promising varieties to be tested in the provinces. In 1999, Low joined the Michigan State University Food Security team in Mozambique’s Ministry of Agriculture. Given that the prevalence rate of vitamin A deficiency (VAD) among children under five years of age in Mozambique was an astronomical 71.2% (Aguayo et al., 2005), the need for improved access to vitamin A rich foods was urgent. She recognized that to convince the global nutrition community, biochemical evidence of impact was required. Low approached twenty-one different donors before she was finally able to get funding^5^ for the *Towards Sustainable Nutrition Improvement (TSNI)* project, based in one of the poorest provinces of Mozambique, Zambézia. Low moved to Zambézia in 2002 to lead the study.

Andrade released nine selected OFSP varieties (bred outside of Africa) in 1999. In addition, Low and Andrade collaborated to raise funds for using OFSP varieties to respond to the devasting floods that occurred in Southern and Central Mozambique in 2000. This was an 18-month long emergency response effort supported by OXFAM during which over 100,000 households received sweetpotato vines. Working with the government’s nutrition division, an innovative demand creation campaign was built around using orange as a color of good health, and the slogan *O Doce que Dá Saúde* (*The Sweet that Gives Health*). It consisted of community theater, performed in each village concurrent with planting material distribution, promotional materials having key messages about OFSP, including *capulanas* (fabric worn as skirts by women), t-shirts, and hats; and radio programs.

Bouis organized a second CGIAR-wide meeting on agriculture and nutrition in 1999 to present research progress. A special edition of the conference proceedings was published in the Food and Nutrition journal in 2000, which included a paper on the OFSP work conducted earlier in Kenya. Substantial support from several major donors was obtained, and Phase 1 of the HarvestPlus program, led by IFPRI and CIAT was launched in 2003.

When Mwanga returned to Uganda from his doctoral studies, breeding for high beta-carotene was included as trait of importance in the national sweetpotato breeding program.

*Organizational Dimension.* CIP's efforts to develop OFSP in Africa continued on a small scale until 2000, mostly in Uganda. In 2001, with the goal of having healthy, rural populations through food-based approaches, especially vitamin A, CIP launched the Vitamin A for Africa (VITAA) Partnership to engage national programs and NGOs from Kenya, Ethiopia, South Africa, Tanzania and Uganda. However, the VITAA Partnership initiative was not able to raise substantial funding and HarvestPlus supported CIP’s sweetpotato breeding efforts and annual VITAA meetings from 2003 through 2008.

Meanwhile in Mozambique, the success of the emergency project led USAID and the government of Mozambique to support Andrade, still with IITA, to disseminate OFSP in other provinces of Mozambique from 2002 through 2006.

*Technological Dimension.* The TSNI project was an opportunity to study in-depth the integrated agriculture-nutrition education-marketing approach (Low et al., 2007). The 18-month study compared 498 mother-child pairs who received the integrated intervention (“intervention households”) to 243 mother-child pairs from areas where no intervention was made (“control households”). The agricultural component consisted of multiplying and distributing OFSP varieties. The nutrition component focused on empowering women and men to adopt improved household diets and women, in particular, to improve young child feeding and hygiene practices. A demand creation campaign also supported the marketing component. By the end of the study, vitamin A intakes among intervention children (median value of 426 μg of retinol activity equivalents (RAE) daily) were significantly higher than those among control children (56 μg RAE). Controlling for infection and other confounders, a 15% decline in the prevalence of VAD was attributable to the integrated intervention (Low et al., 2007b).

Many food scientists in the region had been promoting the use of sweetpotato flour with limited uptake for years, probably due to its high cost. Another innovation component that was added during the TSNI was based on Low’s realization that it was more technically and economically viable to use steamed and mashed OFSP (purée) than dried chips or flour to partially substitute wheat flour in bread *and* the puree-based products tasted better (Low and van Jaarsveld, 2008).

Concurrently, HarvestPlus financed an efficacy trial among school children (5–10 years old) in South Africa (van van Jaarsveld et al., 2005), which controlled the amount of sweetpotato served to school children for 53 days. Vitamin A status was determined using a modified-relative dose response test. The treatment group (n=90) consumed 125 g of boiled, mashed OFSP daily, while the control group (n=90) received the equivalent amount of white-fleshed sweetpotato. The treatment group showed a significant improvement in the amount of vitamin A stored in the liver, compared to the control group.

These two studies were among the first solid evidence that a biofortified crop could contribute to improved micronutrient status, under controlled conditions at a school and in a community setting where households decide whether to produce or consume the crop.

Concerning breeding, CIP had incorporated some local landraces from SSA as parents into its headquarters breeding program in 1998. However, when the advanced clones from that program arrived in Uganda for testing in 2002, within a year almost all had collapsed due to Sweet Potato Virus Disease (SPVD). The bottom line is that virus pressure in East Africa is extremely high and even clones doing well in relatively high virus pressure areas of Peru did not make it.

Moreover, in Mozambique, although the OFSP varieties in the TSNI effort out yielded local landraces and their taste was acceptable to consumers, the varieties struggled to survive with a longer dry season than in central Uganda. Farmers traditionally leave roots in the ground during the dry season to sprout when the rains start again; then multiply these sprouts to get enough material to plant a sweetpotato crop. The foreign-bred materials did not sprout well after being left in the ground. Andrade and Low recognized that resources needed to be raised to breed in Mozambique, by combining the best OFSP introduced varieties with more drought-tolerant local landraces that have key traits demanded by farmers. Convinced by the evidence, the Rockefeller Foundation agreed in 2005 to begin supporting such an effort for breeding in Mozambique.

By 2005, the evidence had also convinced CIP of the need for more decentralized breeding efforts. The new global head of breeding recognized that advantage could be taken of the fact that sweetpotato was propagated by vines. Instead of starting out with one large observational trial in one site, multiple copies could be made of the cuttings to be planted, and 2–3 sites could be used in the 1^st^ season including a stress site of interest, for example, with high drought pressure. The Accelerated Breeding Scheme (ABS) reduced varietal development time from 8–9 years to 4–5 years (Grüneberg et al., 2015), a major complementary innovation. In SSA, Andrade began testing the ABS approach in 2006.

*Status of scaling.* Activities were focused on varietal testing and proof-of concept studies, with less than 15,000 households being reached in Kenya, Uganda and Mozambique. However, in Mozambique, approximately 102,000 were reached through the flood-response program; followed by a large-scale dissemination effort that reached over a million households with selected OFSP varieties.

### 3.3 Phase 3: Evaluation of the Potential to Scale Cost-Effectively (2006–2009)

#### 3.3.1 Institutional environment dimension

The 2007–2008 food price crisis in grains brought agriculture back to the forefront globally (The World Bank, 2007). The Bill & Melinda Gates Foundation (BMGF) began heavily investing in agriculture in 2006. BMGF had already seen the potential for investing into biofortification but were keen to have evidence at scale.

Additionally, interest in nutrition was also gaining steam, reflected in the publication in 2008 of articles on maternal and child nutrition in the influential journal, the *Lancet*. These findings led to increased focus on community-level interventions during the first 1000 days, that is among pregnant women, children less than 24 months of age, and their mothers, because of the lasting impact that undernutrition during the first 1000 days has on future nutritional status, cognitive ability, and overall health (The World Bank, 2014).

#### 3.3.2 Leadership dimension

Bouis leveraged funds from BMGF to undertake a randomized, controlled effectiveness trial (RCT) to evaluate whether the integrated agriculture-nutrition model using OFSP as the key entry point could be taken to scale at reasonable cost in Mozambique and Uganda. Known as the Reaching End Users (REU) study, scale in this context meant improving vitamin A intakes in 14,000 households in four districts in Zambézia Province, Mozambique and 10,000 households in three districts of Uganda (Arimond et al., 2010). In Uganda, Mwanga released the first OFSP varieties, Kabode and Vita, that had moderate virus resistance and were included along with two OFSP local landraces in the REU intervention in that country. Kabode was to subsequently be released in Kenya, Tanzania, Ethiopia, Rwanda, and Burundi, demonstrating its wide adaptability in the sub-region.

Low returned to CIP as Regional Director for Africa in 2005. Andrade joined CIP in 2006, continuing to work in Mozambique, but full-time on OFSP. The orange branding approached used in Mozambique was adopted at the regional level. By the end of 2009, resources had been raised to support CIP-led projects developing and promoting OFSP in 10 SSA countries.

As the positive findings from the REU project began to emerge, Low realized that unleashing the potential of sweetpotato to truly serve smallholder African families would require more local breeding of adapted varieties, addressing key bottlenecks in the production system, especially the seed system, and working with a range of disciplines and partners. In 2008, BMGF financed CIP to engage with a broad range of stakeholders in researching the gaps in knowledge concerning sweetpotato (Andrade et al., 2009) and to design a five-year action research project with selected partners known as Sweetpotato Action for Security and Health in Africa (SASHA) project.

*Technological Dimension.* HarvestPlus led the REU effectiveness trial, with IFPRI conducting the impact study. CIP was responsible for seed systems research and the Natural Resources Institute for the marketing research, with several agriculture and nutrition NGOs as implementation partners. The integrated model used in the TSNI study was adapted to have two different levels of intensity of extension contact for the nutrition education component. In both countries, vitamin A intakes increased significantly among both women and their young children, with OFSP contributing 78% of total vitamin A intake among children 6–35 months of age in Mozambique and 53% in Uganda. Significantly, the less costly, less intensive model was just as effective as the more intense intervention (de Brauw et al., 2018). Vitamin A status of young children was only measured in Uganda and was significantly higher among intervention than control children (Hotz et al., 2012). Average cost per beneficiary (direct and indirect) household that adopted OFSP was $153/household in Mozambique and $100/household in more densely populated Uganda (de Brauw et al., 2018). The intervention was found to be highly cost-effective per disability-adjusted life years saved (Arimond et al., 2010). These findings provided convincing evidence to the global nutrition community that biofortified OFSP could work at scale.

The REU project also tested different models of “seed” dissemination. Sweetpotato is propagated in the tropics through cuttings from vines. These materials are perishable and easily shared among farmers. Unlike hybrid maize, private sector seed companies have limited interest in sweetpotato. However, yield-reducing high virus pressure conditions in bimodal rainfall areas (found in Uganda), and prolonged dry conditions in unimodal areas (Mozambique and Uganda) makes the seed system a critical component of the OFSP innovation package. The model of free mass dissemination to farmers gathered in villages was the principal strategy used in Uganda; in Mozambique, networks of decentralized vine multipliers were established to reduce the distance between farmers and the source of vines.

The prolonged dry season challenge led to an innovation based on traditional methods for assuring planting material after the long dry season by leaving some roots in the ground at harvest to sprout when the rains started. The problem is that these roots often became weevil infested and the amount of planting material produced was limited. The innovation developed consisted of storing healthy, small to medium size roots in a storage container alternating with layers of sand for 4–6 months. Six to eight weeks before the rains are due to start, the stored (and by then sprouted) roots are planted out in a protected seed bed. On average, 40 cuttings are obtained per root by the time the rains start (Namanda et al., 2013). This innovative seed system technology was named “Triple S”, that is Storage in Sand, then Sprouting.

#### 3.3.3 Organizational dimension

By 2009, CIP had clearly mainstreamed breeding for OFSP as its flagship product and OFSP promotion became a dominant component in their communication strategy. Henceforth, CIP took the major lead in the development and promotion of OFSP, while HarvestPlus focused its research efforts on the other biofortified crops, while continuing to promote OFSP in Uganda as a follow-up effort to the REU investment.

In 2008, Low approached the Alliance for Green Revolution in Africa (AGRA) to seek their support in contributing to building a critical mass of sweetpotato breeders as they were investing in doctoral training programs in South Africa and Ghana. This subsequent six-year collaboration established a core group of sweetpotato breeders based in national programs.

*Status of scaling.* By the end of this phase, the number of households reached, registered with quality dissemination data were 14,000 in Mozambique, 10,000 in Uganda and 376 in Kenya and Tanzania. However, 2009 saw the initiation of new OFSP scaling projects in Ethiopia, Malawi, Angola, and Mozambique. To our knowledge, no major non-project OFSP dissemination efforts existed.

### 3.4 Phase 4: Significant investment in research to address breeding and other bottlenecks initiated and launching of Sweetpotato for Profit and Health Initiative (2010–2014)

#### 3.4.1 Institutional environment dimension

This is a critical period when agriculture and nutrition truly began to align. First, the global Scaling Up Nutrition (SUN) movement began in 2010, as an advocacy platform that encourages countries to commit to a set of nutrition targets, with a heavy emphasis on reducing child stunting, achieved by implementing nutrition-specific and nutrition-sensitive interventions^6^. The nutrition-sensitive interventions include integrated agriculture-nutrition programs, captured in detail in another influential *Lancet* series (Ruel et al., 2013). Reviews called for more evidence of impact from food-based interventions, noting that OFSP-focused interventions had the best evidence base to date (Ruel and Levin, 2000; Girard et al., 2012).

DFID (UKAid), Irish Aid, and USAID became vocal supporters of nutrition and hunger efforts. A major Global Nutrition for Growth event, sponsored by the United Kingdom, was held in 2013. Subsequently, DFID provided substantial funding for OFSP scaling in four countries and USAID included OFSP as a component in several of its Feed the Future country initiatives. Moreover, donors supported a series of multi-sectoral, sub-regional planning meetings to integrate nutrition-sensitive activities into CAADP planning processes during 2012–2013.

#### 3.4.2 Organizational dimension

When BMGF awarded CIP the SASHA project in 2009, it was the largest investment in sweetpotato research in SSA ever made ($22.5 million dollars). SASHA focused on using the accelerated breeding scheme (ABS), as adapted varieties were the core innovation supported by seed systems research, delivery system research, and strengthening the sweetpotato community of practice.

This support enabled CIP to concurrently launch the multi-donor, multi-partner Sweetpotato for Profit and Health Initiative (SPHI) with the goal to reach 10 million SSA households in 17 target countries by 2020 with improved varieties of sweetpotato and their diversified use (Low, 2011). The SPHI was a vital institutional innovation for scaling, aligning five donors: BMGF, USAID, DFID (UKAid), Irish Aid, and AGRA. The SPHI Steering Committee included the major donors, partners(including HarvestPlus), and dissemination stakeholders. BMGF supported research and proof-of-concept studies; AGRA national program breeding and seed system work; with USAID, DFID and Irish Aid focused on dissemination. It was envisioned that SASHA and AGRA investments in breeding during the first five years would provide the adapted varieties necessary for increased scaling efforts during the second phase (2015–2019). Non-governmental organizations were requested to attend the annual technical meeting and annually submit data on the numbers of direct and indirect beneficiary households reached with improved sweetpotato varieties, using an agreed upon definitions of beneficiary types^7^.

#### 3.4.3 Leadership dimension

Low became the project manager of SASHA and leader of the SPHI for CIP. Mwanga joined CIP as the sweetpotato breeder for East and Central Africa. Andrade continued to lead CIP’s Southern African breeding program, releasing 15 drought-tolerant varieties bred in Mozambique using the ABS in 2011 (Andrade et al., 2016).

#### 3.4.4 Technological dimension

The massive investment in sweetpotato breeding in Africa, with CIP leading three sub-regional population development programs in Mozambique, Uganda, and Ghana and backstopping nine national sweetpotato breeding programs financed by AGRA, was critical for subsequent scaling with adapted OFSP varieties that met the taste and other preferences of consumers. Uptake of ABS by national partners resulted in the release of 40 OFSP varieties bred in Africa, in 9 countries during this period.

In high virus pressure areas, a complementary innovation was developed for bi-modal rainfall areas known to have high “virus-pressure”. These *net tunnels* (a 3.6 square meter structure covered by horticultural netting) were designed to prevent insects from infecting the disease-free stock of pre-basic planting material that the trained vine multipliers received from national programs. The basic principle was that if multipliers could maintain their own disease-free foundation seed, the need to return to the national research station to replenish their seed would diminish. The root-based Triple S technology for drought-prone areas was also successfully validated during this period.

As part of SASHA, in collaboration with the international NGO Catholic Relief Services and many national NGOs in Tanzania, a major scaling-effort of disease-free seed was undertaken in Western Tanzania. This revealed the pros and cons of utilizing mass dissemination versus setting up networks of community-level decentralized vine multipliers and the challenge of monitoring dissemination efforts across numerous local organizations with different capacities (McEwan et al., 2017). In addition, operations research led to better strategies to ensure that women participated as vine multipliers (Badstue and Adam, 2011).

Innovation continued in terms of delivery models as well. The first, referred to a Mama SASHA, linked OFSP vine access to ante-natal care (ANC) health services for pregnant women (Cole et al., 2016). In addition, nutrition education was incorporated into ANC counseling sessions and reinforced at monthly, community level pregnant women’s clubs. This model aligned with the focus of the nutrition community on targeting the first 1000 days, i.e. pregnancy and the first two years of an infant’s life, as the critical time for young child growth and cognitive development (Girard et al., 2017).

The second delivery system built on prior proof-of-concept work that used OFSP purée to partially replace wheat flour in a baked product. Collaborating with the largest agro-processing company in Rwanda over four years, a successful gender-sensitive value chain was developed for OFSP roots supplied for processing an OFSP biscuit and donuts (Ndirigwe et al., 2015). This success helped change the image of sweetpotato with the government, who began to see its potential as a cash crop and healthy food. This government subsequently included OFSP promotion as part of its nutrition strategy.

*Status of scaling.* For scaling to occur, regional and national government support is required. BMGF again was at the forefront of enabling CIP and the nutrition-focused NGO Helen Keller International (HKI) to identify and train advocates for biofortification and OFSP in particular, as well as develop training programs for technical change agents. The *Reaching Agents of Change* (RAC) investment lasted three years (2011–2013) and focused on four countries: Mozambique, Tanzania, Nigeria, and Ghana. This led to significant investments in OFSP dissemination by district governments in Tanzania and federal agencies in Nigeria and Mozambique. CIP, HKI, and HarvestPlus staff, along with the trained advocates, strove to get biofortification included in food security, nutrition, micronutrient, and agricultural development strategies with considerable success at the regional and country level (Covic et al., 2017).

An innovative ten-day learning by doing *Everything you ever wanted to know about sweetpotato* trainer-of-trainers course was developed by CIP and the Natural Resources Institute in four languages^8^. Local training institutions in Mozambique, Tanzania, Nigeria, Ghana, and Burkina Faso were strengthened to conduct this course between 2012 and 2015.

With the launching of the SPHI, more systematic data collection on annual dissemination of planting material of improved sweetpotato varieties began to take place. From 2010–2014, 1.13 million households were reached across nine SSA countries, encompassing direct and indirect beneficiaries. Non-CIP NGO partners contributing to the SPHI effort included HarvestPlus, Farm Concern International, and HKI. CIP, HarvestPlus, and HKI emphasized approaches focused on improved nutrition outcomes, whereas Farm Concern concentrated on market development and income generation.

### 3.5 Phase 5: Significant scaling under the Sweetpotato for Profit and Health Initiative (2015-mid-2019)

#### 3.5.1 Institutional environment dimension

At the beginning of this period, the value of integrated agriculture-nutrition interventions had been clearly recognized by the international nutrition and agriculture communities, along with major donors and many SSA governments. Since 2015, there has been increasing emphasis on working to improve whole food systems rather than focusing on single nutrients (Mozaffarian et al., 2018). OFSP is well-positioned within the emerging food system framework, as it is produced in all major SSA food systems. Sixty-six percent of the area under sweetpotato is grown in the three farming systems where 48% of the rural population of SSA resides^9^.

#### 3.5.2 Organizational environment

BMGF awarded CIP a second five-year phase for SASHA (2014–2019), for $21.6 million. This continued to support the validation of innovative breeding methods to accelerate breeding and exploit hybrid vigor, included a new emphasis on working with local institutions to ensure sustained production of early generation seed, and added post-harvest research focused on improving the promising OFSP purée by developing a vacuum-packed, shelf-stable product and solar powered cold storage.

The membership, particularly among NGOs on the SPHI Steering Committee expanded. In 2018, members of the SPHI Steering Committee consisted of five donors, six NGOs, two international research universities, two CGIAR programs (Roots, Tubers and Bananas and HarvestPlus), one private sector company, and the co-leading organization, the Forum for Agricultural Research in Africa (FARA). Donor support for dissemination continued to be strong from DFID, USAID and Irish Aid, with EU becoming a significant contributor as well. One core contribution of SPHI is its use of common indicators across partner organizations, critical for coherently monitoring scaling progress.

AGRA discontinued new support for national program breeding efforts due to a change in their strategic direction. By 2018 the number of doctoral-level sweetpotato breeders in Africa had increased to 22 in 2018 from 8 in 2005, mostly driven by AGRA support.

By the end of 2018, over 5,600 change agents had been trained through the Trainer of Trainer’s system in the five countries. Most participants were government extension and NGO personnel.

CIP joined with FARA to continue to promote the integration of biofortification into regional and national food security and nutrition strategies. HarvestPlus also engaged heavily in advocacy. Considerable progress has been made (Covic et al., 2017), with biofortification and/or nutritious foods integrated into 7 regional strategies, 23 national agriculture strategies, and 18 national nutrition strategies in SSA between 2011–2019.

*Technological Dimension.* From 2009 through mid-2019, a total of 93 OFSP varieties were released in 16 SSA countries; 74 of these bred by 12 SSA breeding programs. The Triple S seed innovation has been validated in 8 countries and is beginning to scale significantly in Uganda, Ethiopia, and Ghana, with support from the CGIAR Research Program on Roots, Tubers, and Bananas to test ways to enhance the scaling process. The net tunnel technology has proven to be a challenge for smaller-scale vine multipliers to maintain and now larger mini-screenhouse units are recommended for sub-stations and larger basic seed multipliers with enough management skill.

Considerable investment has been made to develop institutional and technical conditions for 11 public sector research institutions to develop and implement business plans for pre-basic seed production, with rotation funds being the mechanism for sustaining critical pre-basic seed production when SASHA Phase 2 closes in 2019 (Rajendran et al., 2017).

The use of OFSP purée continues to expand, with a private sector factory in Kenya producing the product as a functional ingredient sold to two major supermarkets, who in turn are producing OFSP bread on a regular basis in their stores in the capital city (Bocher et al., 2017).

Innovation concerning delivery systems continued. In Nigeria OFSP porridge was successfully introduced into a school feeding program in Osun State. In Uganda, OFSP school books linked to vine access were introduced into the curriculum of 55 primary schools. In Ethiopia, an improved integrated agriculture-nutrition component at the community level is underway with the introduction of a Healthy Toolkit, consisting of a bowl indicating quantities of porridge to be served at different age periods for children and a slotted spoon to ensure sufficient porridge thickness, to complement community level nutrition training. These are proof-of-concept efforts where evaluations accompany their introduction to build the evidence base.

Promotion of the orange brand continues, with greater use of social media enhancing awareness creation, especially among youth. Digital tools such as WhatsApp are helping to improve coordination in seed delivery.

#### 3.5.3 Leadership dimension

In recognition of their efforts to make biofortification a reality, the 2016 World Food Prize was awarded to Low, Andrade, Mwanga and Bouis. This has enhanced visibility of the effort, both globally and in SSA, but increased the pressure to scale even faster.

**Fig. 1:**
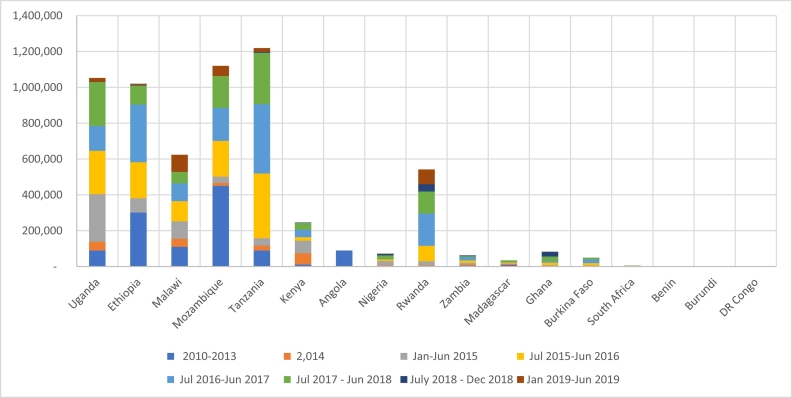
Number of households reached with improved varieties of sweetpotato by country (2009-mid-2019).

*Status of scaling.* An additional 5.0 million households were reached in 12 countries during this period. This means as of July 2019, 6.2 million SSA households had been reached under the auspices of the SPHI (Fig. 1) (Okello et al., 2019). The degree of going-to-scale varies greatly between the 16 SPHI target countries, largely reflecting donor priorities in target country choice combined with investment in breeding. In each of six countries (Tanzania, Uganda, Malawi, Mozambique, Kenya, Rwanda, and Ethiopia) over 200,000 beneficiary households have received improved sweetpotato varieties (Fig. 1). The NGO partners during this phase expanded to include Catholic Relief Services, Farm Africa, and World Vision Australia.

In 2016, a follow-up study to the *Reaching End Users* effectiveness trial was published. Three districts in Uganda were visited in 2011, two years after project support finished. In two of the districts, significant percentage of households were continuing to grow OFSP, 49% in Kamuli and 59% in Mukono, whereas only 8% in Bukedea were still growing. Apparently in Bukedea, a rice promotion project entered the district at the end of the OFSP intervention. The most important determinant of adoption was whether the household had been growing sweetpotato at the start of the intervention (McNiven et al., 2016).

In Mozambique, a nationally representative agricultural survey in 2015 established that orange-fleshed sweetpotato now constituted 32% of all sweetpotato grown in the country (Ministério da Agricultura e Segurança Alimentar, 2015). Dissemination efforts to date have been concentrated in 6 of the countries’ 10 provinces, with the OFSP varieties Irene and Delvia emerging as the most broadly adapted.

## 4 Discussion

Clearly, the scaling process of the OFSP innovation is still underway and at different stages in different countries. In this section, key research questions will be addressed.

### 4.1 What is the role of “leadership” in driving an innovation process?

Continued and committed leadership for innovation is key. Rogers (2003) defines a *champion* as “a charismatic individual who throws his or her weight behind an innovation, thus overcoming indifference or resistance that the new idea may provoke in an organization”. The OFSP story is one of many committed individuals, but particularly four scientists who worked for almost 25 years to promote a new way of breeding and utilizing nutritious crops that often called into question conventional wisdoms. Once recognition is obtained, and the innovative idea is integrated into the organizational home, continued leadership is required to recognize where further research is needed to overcome barriers to scaling.

In the context of scaling, the leaders set clear, but simple visions needed to reach large numbers of beneficiaries and worked on identifying which partners are appropriate for scaling. Setting the SPHI Vision of reaching 10 million households by 2020 with improved varieties of sweetpotato and their diversified use, was an ambitious goal given conditions in 2009. It served, however, as an easy to remember and uniting vision across diverse organizations because it did not dictate how that goal would be reached by each partner.

### 4.2 What is the role of evidence in scaling?

The evidence base for OFSP is often cited as one of the strongest among all food-based approaches (Girard et al., 2012; Ruel et al., 2013). That strong evidence base was central for generating donor investment in scaling and convincing policy makers to prioritize biofortification. Evidence of the cost-effectiveness of the OFSP-focused integrated agriculture-nutrition approach was essential for securing the large investment in the further development and promotion of OFSP and other biofortified crops. Because the proposed innovation was with an “orphan” crop, grown primarily by poor women, and the innovation package required collaboration across disciplines, it took twelve years to generate a progressively stronger evidence base that an OFSP-focused, integrated agriculture-nutrition-market innovation could combat vitamin A deficiency in SSA at scale.

When many partners are involved in scaling, creating agreed upon mechanisms for monitoring, learning, and accountability is essential for continued investment. The Monitoring, Learning and Evaluation Working Group under the SPHI developed a standard set of modules and analytic tools for capturing dissemination, measuring yields through crop cuts, and key dietary quality and sweetpotato knowledge and performance indicators. NGOs who are members of the SPHI Steering Committee submit updated beneficiary data concerning vine dissemination efforts annually. This facilitates the community of practice communicating with a unified voice concerning progress to the donor community.

### 4.3 What are the critical inflexion points in a long-time scale innovation process?

**Fig. 2:**
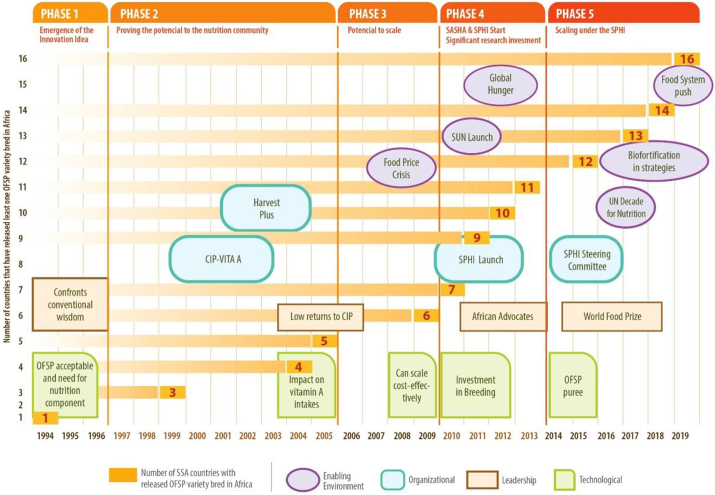
Major inflection points along the OFSP innovation pathway by dimension.

A inflection point has been described as an “event that changes the way we think or act” (Phillips et al., 2016) and if we fail to adjust appropriately to that event, major damage will be done to the organization. Fig. 2 captures key elements of the different technical, organizational, institutional environment, and leadership dimensions that underlie where major “inflection” points occurred along the OFSP innovation process. The first major inflection point occurred in 2005, when the results of the TSNI study demonstrated the impact of the integrated agriculture-nutrition intervention on young child vitamin A intakes and status in central Mozambique. Positive results from the REU study contributed to the second major inflection point in 2009–2010 supported by institutional environment factors: the food price crisis and the launch of the scaling up nutrition movement. The subsequent major investment in sweetpotato research in 2010 resulted in the rapid rise from that point forward (shown by the orange boxes) in the number of SSA countries with at least one released OFSP variety bred in Africa. OFSP varieties adapted to local agronomic conditions *and* local consumer taste preferences were essential for adoption. Concurrently, there was increased investment in OFSP dissemination. It remains to be seen where the next inflection point will occur. CIP considers the use of OFSP purée for partial wheat flour substitution and in other products to be a potential gamechanger. In addition, climate change will likely necessitate greater use of resilient, fast growing crops like sweetpotato, whose leaves as well as roots can be consumed.

### 4.4 What is the role of the public sector in scaling?

OFSP is a vegetatively propagated crop with a visible trait. It is a renowned food security crop in SSA, known as the being the crop that is there when the maize fails. Private sector seed companies have little interest in sweetpotato, as it is easily shared among farmers, or a farmer just needs to buy a small amount of quality planting material and she can multiply it herself. Willingness to pay-for-seed occurs after a long drought (all neighbors have lost material) or when there is a strong market demand for a particular variety and farmers want to maximize yields by using quality seed. In most SSA countries public sector extension systems are underfinanced. Thus, two major models have emerged, both which require the involvement of the public sector. First, is to undertake mass distribution campaigns every 4–5 years as new varieties come on-line and encourage the maximization of farmer-to-farmer exchange. Second, is to establish networks of trained vine multipliers linked to public sector pre-basic seed institutions. The clients of these vine multipliers will be farmers within a reasonable radius and NGOs and government institutions whose principle objectives are improving the nutritional status and food security of the population. Ideally, these vine multipliers will also be commercial root producers so that they mitigate their risk in the face of fluctuating vine demand.

In countries where public sector extension services exist, getting the crop integrated into the national or district level extension service training centers and programs is central. This is underway in parts of Tanzania and Ethiopia. When there is clear market demand, either from school feeding programs or agro-processors requiring consistent supply, that also stimulates other cultivators and actors along the value chain to participate. Farmer-to-farmer sharing of planting material is an integral part of scaling. This requires the released OFSP variety to be a good performer with acceptable taste and in drought-prone areas, be capable of sprouting easily after the dry season. In our experience, farmer-to-farmer movement occurs quickly in densely-populated countries like Rwanda and Malawi.

Clearly, the development of strong root markets in urban areas (which is occurring as urbanization continues to increase) and markets for agro-processed products using OFSP as a key ingredient will drive demand in the long-run. But full-blown scaling of OFSP under such conditions will take longer than for most grain crops. Increasingly, however, urban consumers are becoming health conscious, in part due to the growing diabetes problem. Sweetpotato roots have good levels of dietary fiber, and a medium glycemic index (Bovell-Benjamin, 2010). Moreover, the increasing use of digital technologies to link actors along the value chain, and the growing interest in nutritious foods will continue to increase efficiencies along the value chain.

### 4.5 What are the key elements of the institutional environment in supporting scaling?

Investing in promotion and advocacy to ensure a supportive institutional environment is critical. Building a cadre of within country and regional advocates proved critical for getting sustained commitment and local buy-in to the concept of biofortification by regional bodies and governments, which in turn builds within country ownership and the willingness of donors to invest. Development of demand creation campaigns based on the orange color proved invaluable to getting OFSP recognized as a wise investment. Having training courses available in local institutions^10^ representing the major languages of the continent facilitated having qualified change agents available for the scaling effort. Knowing when government planning exercises are undertaken and who are the key players in determining priorities for resources is critical for moving from integration of biofortification as an objective in national and regional strategies to committing resources and getting actual implementation on the ground.

The scaling process is uneven across partners and countries. There are many different reasons why countries are prioritized for investment by public or private sector entities. Consequently, there is great unevenness as to the degree and type of scaling underway in SSA, which can cause confusion among those not fully aware of all of the steps required for conditions for scaling to be in place. Guidelines for how to design, cost out, and implement an integrated intervention were developed to assist new entries (Stathers et al., 2015).

### 4.6 What new research needs appear and are addressed as scaling happens?

Once scaling starts, research does not end as new bottlenecks emerge or country specific adaptation is required. There is no one-size-fits-all model for the diverse and complex conditions found in SSA. Investments in operations/formative research to adapt the integrated agriculture-nutrition model and develop innovative delivery systems is requisite to ensure uptake. The emergence of agro-processing led breeders to pay more attention to root shape, skin color, and starch and sugar composition. By studying which improved varieties are spreading most, breeders are learning the traits which are essential for different agro-ecologies. Creating operational sweetpotato seed systems at scale is *the* major challenge, as these systems are very underdeveloped at the start in most SSA countries.

## 5 Conclusion

Much has been learned about scaling from the OFSP research for development investment to date. Woltering et al. (2019) argue that pilot projects managed under controlled conditions rarely scale well. However, the research linked to pilot integrated agriculture-nutrition-marketing efforts in the OFSP case were critical for generating evidence to convince donors to invest in biofortification. Woltering et al. (2019) also call for scaling to be treated as a transformation process that helps brings the necessary organizational and institutional arrangements into place, so that true impact at scale can be achieved. The OFSP case illustrates how such a transformation process played out across four key dimensions.

Hence in addition to the **technical** dimension around the core varietal innovation, the presence of the other three dimensions was critical in each of the five phases described. In terms of **organization**, the International Potato Center was willing to go beyond its traditional mandate of breeding and applied research, to facilitation of dissemination in collaboration with partners. Learning how to scale effectively became a major theme of the CGIAR Research Program on Roots, Tubers, and Bananas, which CIP leads. The **leadership** dimension was particularly important at the outset and at the point of raising resources for scale. Leadership combined with the institutional environment drove organizations to adapt and change. The best way to learn and feedback findings, including how different varieties are performing, into research formulation is to be engaged in the scaling process itself. Finally, for the **institutional** dimension the positive environment was fortuitous and mostly beyond the control of those engaged in the OFSP effort; nevertheless, the strong evidence base for the nutritional impact of integrated agriculture-nutrition interventions using OFSP as a key entry point did contribute to willingness of donors and governments to take on nutrition-sensitive approaches.

Within the technical dimension, many complementary innovations, especially in seed system management and delivery systems, were developed to ensure that released OFSP varieties would be able to make their intended impact. Innovations were adapted to whether sweetpotato is being produced in high virus pressure bimodal rainfall areas, or more drought-prone unimodal rainfall zones. An array of different gender-responsive delivery systems were designed according to whether the primary desired outcome was improved food security and nutritional status or income generation.

It has taken 25 years to come this far, like many other successful agricultural interventions (Rogers, 2003). But the large investment in breeding adapted orange-fleshed sweetpotato varieties in Africa has really only taken place during the past decade. Progress in breeding methods and investment in training national breeders has transformed sweetpotato breeding in Africa, making it faster and better. Over 6 million households have been reached with improved varieties of orange-fleshed sweetpotato. However, given the time lag to adoption, the true impact of the considerable investment in *breeding sweetpotato in Africa for Africa* will need to wait at least another five years to truly assess.

Looking to the future, investment in building cadres of vine-root enterprises linked to sources of well managed pre-basic seed continues to be needed. More attention should be given to addressing the needs and preferences of urban consumers. Hence exploring the willingness of governments to incentivize use of OFSP purée as a functional ingredient in processed products looks to be a win-win strategy for a substantial change in food systems overly reliant on imported staples.

Sustained investment will be needed to ensure the technological and institutional environments are supported so that the scaling up effort of OFSP does not stall. The value of the intervention has been demonstrated. Further innovation is required to unleash the full potential of the technology on the continent. Certainly, during the past decade, the institutional environment for OFSP as an innovation package has changed, with a recognition that more nutritious foods must be in the diet and regional bodies and many national governments explicitly acknowledging biofortification as a cost-effective intervention. In most countries, sweetpotato is no longer being referred to as an “orphan crop”. Most African governments, however, still do not meet their targets for support to the agriculture sector. But in countries that are investing more in agriculture, and with the progress made integrating biofortification into national and regional government policy, the commitment of public resources to nutritious crops like OFSP will gradually increase, as we are seeing in countries like Tanzania and Ethiopia, as part of developing healthier, more sustainable food systems.

## Declarations of Competing Interest

none.

## Footnotes

1 The concept of disruptive innovation is widely used in business circles. It was coined by Bower and Christensen (1995) who explain: ”Disruptive innovation often sacrifices performance along dimensions that are important to current customers and offers a very different package of attributes that are not (yet) valued by those customers. At the same time, the new attributes can open up entirely new markets”.

2 In East Africa it is becoming increasingly a cash crop, driven by increased awareness of health benefits and challenges in the food sector, which have increased its competitivity compared to maize.

3 The first OFSP variety bred in Uganda, NASPOT 5, was released in 1999, but proved be unstable in performance across different agro-ecologies. However, it became an important parent in the polycross breeding program during the first decade of the 21^st^ century.

4 A term only coined in 2001.

5 The major donor was the Micronutrient Initiative of Canada. Also contributing were the Rockefeller Foundation, USAID-Washington, and during the final year HarvestPlus.

6 Nutrition-specific interventions include promoting breastfeeding, micronutrient supplementation, complementary feeding education, etc. Nutrition-sensitive activities involve engaging with other sectors, such as agriculture or education, to promote nutrient-dense foods and include nutrition education in school curricula.

7 A direct beneficiary was defined as receiving vines of improved varieties from a known source (i.e. project or program); indirect beneficiaries received vines of improved varieties from other farmers or market purchase. The number of indirect beneficiaries was often based on project policy (i.e. you are expected to share vines the next season with 2 neighbors, for example) or from data gathered on vine sharing or sales in structured household surveys.

8 The training course development and advocacy work was financed by the Bill and Melinda Gates Foundation as part of the Reaching Agents of Change project.

9 The three farming systems are root crop, mixed cereal-root crop, and mixed maize (You et al. 2005). Population data from Rose, Amy N., McKee, Jacob J., Urban, Marie L., Bright, Eddie A. 2018. LandScan 2017. Oak Ridge. Oak Ridge National Laboratory.

10 All training materials are available on the Sweetpotato Knowledge Portal: www.sweetpotatoknowledge.org.

## References

[bb0005] Aguayo V.M., Kahn S., Ismael C., Meershoek S. (2005). Vitamin A deficiency and child mortality in Mozambique. Public Health Nutr..

[bb3000] Andrade M., Barker I., Cole D., Dapaah H., Elliott H., Fuentes S., Grüneberg W., Kapinga R., Kroschel J., Labarta R., Lemaga B., Loechl C., Low J., Lynam J., Mwanga R., Ortiz O., Oswald A., Thiele G., Barker C. (2009). Unleashing the potential of sweetpotato in SubSaharan Africa: Current challenges and way forward.

[bb0010] Andrade M.I., Ricardo J., Naico A., Alvaro A., Makunde G.S., Low J.W., Ortiz R., Gruneberg W.J. (2016). Release of orange-fleshed sweetpotato (*Ipomoea batatas* [l.] Lam.) cultivars in Mozambique through an accelerated breeding scheme. J. Agric. Sci..

[bb0015] Arimond M., Ball A.-M., Béchoff A., Bosch D., Bouis H., de Brauw A., Coote C., Dove R., Eozenou P., Gilligan D., Hotz C., Kumara N., Labarta R., Loechl C., Low J., Magezi S., Massingue J., Meenakshi J.V., Moursi M., Musoke C., Namanda S., Nsubuga H., Okwadi J., Tomlins K., Wamaniala M., Westby A. (2010). Reaching and engaging end-users (REU) with orange fleshed sweet potato (OFSP) in East and Southern Africa. Final report submitted to the Bill & Melinda Gates Foundation.

[bb0020] Badstue L., Adam R. (2011). Gender and vines: Production, management and exchange of sweetpotato planting material among smallholders in the Lake Victoria region, Tanzania.

[bb0025] Bocher T., Low J.W., Muoki P., Magnaghi A., Muzhingi T. (2017). From lab to life: Making storable orange-fleshed sweetpotato purée a commercial reality. Open Agric..

[bb0030] Bouis H.E. (2002). Plant breeding: a new tool for fighting micronutrient malnutrition. J. Nutr..

[bb7000] Bouis H., Saltzman A., Low J.W., Ball A.-M., Covic N. (2017). Chapter 1: An overview of the landscape and approach for biofortification in Africa.

[bb0035] Bovell-Benjamin A.C., Ray R.C., Tomlins K.I. (2010). Sweet potato utilization in human health, industry and animal feed systems. Sweet Potato: Post harvest aspects in food, feed, and industry.

[bb0040] Bower J.L., Christensen C.M. (1995). Disruptive technologies: catching the wave. Harv. Bus. Rev..

[bb0045] Cole D.C., Loechl C., Levin C., Thiele G., Grant F.K., Girard A.W., Low J.W. (2016). Designing, implementing and evaluating an integrated agriculture and health service intervention to improve nutrition outcomes using bio-fortified sweetpotato in Western Province, Kenya. Eval. Program Plann..

[bb0050] Covic N., Low J.W., MacKenzie A., Ball A.-M. (2017). Advocacy for biofortification: Building stakeholder support, integration into regional and national policies and sustaining momentum. Chapter 16. Special Issue on Biofortification. African Journal of Food, Agriculture, Nutrition and Development.

[bb0055] de Brauw A., Eozenou P., Gilligan D.O., Hotz C., Kumar N., Meenakshi J.V. (2018). Biofortification, crop adoption and health information: Impact pathways in Mozambique and Uganda. Am. J. Agric. Econ..

[bb0060] Feed the Future (2017). The U.S. Government’s Global Food Security Research Strategy: Reducing Global Hunger, Malnutrition and Poverty through Science, Technology and Innovation.

[bb0065] Girard A.W., Self J.L., McAuliffe C., Olude O. (2012). The effects of household food production strategies on the health and nutrition outcomes of women and young children: a systematic review. Paediatr. Perinat. Epidemiol..

[bb0070] Girard A.W., Grant F., Watkinson M., Okuku H.S., Wanjala R., Cole D., Levin C., Low J. (2017). Promotion of orange-fleshed sweet potato increased vitamin A intakes and reduced the odds of low retinol-binding protein among postpartum Kenyan women. J. Nutr..

[bb0075] Gliddon D.G. (2006). Forcasting a competency model for innovation leaders using a modified delphi technique.

[bb0080] Graham R.D., Welch R., Bouis H.E. (2001). Addressing micronutrient malnutrition through enhancing the nutritional quality of staple foods: Principles, perspectives and knowledge gaps. Advances in Agronomy.

[bb0085] Grüneberg W.J., Ma D., Mwanga R.O.M., Carey E.E., Huamani K., Diaz F., Eyzaguirre R., Guaf E., Jusuf M., Karuniawan A., Tjintokohadi K., Song Y.-S., Anil S.R., Hossain M., Rahaman E., Attaluri S., Some K., Afuape S., Adofo K., Lukonge K., Karanja L., Ndirigwe J., Ssemakula G., Agili S., Randrianaivoarivony J.-M., Chiona M., Chipungu F., Laurie Ricardo S.J., Andrade M., Fernandes F.R., Mello A.S., Khan A., Labonte D.R., Yencho G.C. (2015). Chapter 1. Advances in Sweetpotato Breeding from 1993 to 2012. Potato and Sweetpotato in Africa: Transforming the Value Chains for Food and Nutrition Security.

[bb0090] Hagenimana V., Oyunga M.A., Low J., Njoroge S.M., Gichuki S., Kabira J. (1999). The effects of women farmer's adoption of orange-fleshed sweet potatoes: raising vitamin A intake in Kenya. International Center for Research on Women.

[bb0095] Hall A., Rasheed Sulaiman V., Clark N., Yoganand B. (2003). From measuring impact to learning institutional lessons: an innovation systems perspective on improving the management of international agricultural research. Agric. Syst..

[bb0100] Hartmann A., Linn J. (2008). Scaling up: a framework and lessons for development effectiveness from literature and practice. SSRN Electron. J..

[bb0105] HarvestPlus (2018). Our History. https://www.harvestplus.org/about/our-history.

[bb2000] Haskell M.J., Jamil K.M., Hassan F., Peerson M., Hossain M.I., Fuchs G.J., Brown K.H. (2004). Daily consumption of Indian spinach (Basella alba) or sweet potatoes has a positive effect on total body vitamin A stores in Bangadeshi men. Am J Clin Nutr.

[bb0110] Hotz C., Loechl C., Lubowa A., Tumwine J.K., Ndeezi G., Nandutu Masawi A., Baingana R., Carriquiry A., de Brauw A., Meenakshi J.V., Gilligan D.O. (2012). Introduction of beta-carotene-rich orange sweet potato in rural Uganda resulted in increased vitamin A intakes among children and women and improved vitamin A status among children. J. Nutr..

[bb0115] IFAD (2015). IFAD’s operational framework for scaling up results.

[bb0120] International Vitamin A, Consultative Group (IVACG) (1999). The Bioavailability of Dietary Carotenoids: Current Concepts. International Vitamin A Consultative Group (IVACG).

[bb1000] Jalal F., Nesheim M., Agus Z., Sanjur D., Habicht J. (1998). Serum retinol concentrations in children are affected by food sources of B-carotene, fat intake, and anthelmintic drug treatment. Am J Clin Nutr.

[bb4000] Jones K.M., de Brauw A. (2015). Using Agriculture to Improve Child Health: Promoting Orange Sweet Potatoes Reduces Diarrhea. World Development.

[bb0125] Kimenyi M.S., Routman B., Westbury A. (2013). CAADP at 10: Progress Toward Agricultural Prosperity. Africa Growth Initiative Policy Paper.

[bb6000] Laurie S., Faber M., Adebola P., Belete A. (2015). Biofortification of sweet potato for food and nutrition security in South Africa. Food Research International.

[bb5000] Low J., Ball A., Jaarsveld P.J., Namutebi A., Faber M., Grant F.K., Low J. (2015). Assessing Nutritional Value and Changing Behaviours Regarding Orange-fleshed Sweetpotato Use in Sub-Saharan Africa..

[bb0130] Low J.W. (2011). Unleashing the potential of sweet potato to combat poverty and malnutrition in Sub-Saharan Africa through a comprehensive initiative. Acta Hortic..

[bb0135] Low J.W., Preedy V.R., Srirajaskanthan R., Patel V.B. (2013). Biofortified Crops with a Visible Trait: the example of Orange-fleshed Sweetpotato in Sub-Saharan Africa. Handbook of food fortification and health: From concepts to public health applications.

[bb0140] Low J., van Jaarsveld P. (2008). The potential contribution of bread buns fortified with B-carotene-rich sweet potato in Central Mozambique. Food Nutr. Bull..

[bb0145] Low J., Kinyae P., Gichuki S., Oyunga M.A., Hagenimana V., Kabira J. (1997). Combating vitamin A deficiency through the use of sweetpotato: results from phase I of an action research project in South Nyanza, Kenya.

[bb0150] Low J.W., Arimond M., Osman N., Cunguara B., Zano F., Tschirley D. (2007). Ensuring the supply of and creating demand for a biofortified crop with a visible trait: Lessons learned from the introduction of orange-fleshed sweetpotato in drought-prone areas of Mozambique. Food Nutr. Bull..

[bb0155] Low J.W., Arimond M., Osman N., Cunguara B., Zano F., Tschirley D. (2007). A food-based approach introducing orange-fleshed sweet potatoes increased vitamin A intake and serum retinol concentrations in young children in rural Mozambique. J. Nutr..

[bb0160] Low J.W., Lynam J., Lemaga B., Crissman C., Barker I., Thiele G., Namanda S., Wheatley C., Andrade M., Loebenstein G., Thottappilly G. (2009). Sweetpotato in Sub-Saharan Africa. The Sweetpotato.

[bb0165] McEwan M.A., Lusheshanija D., Shikuku Kelvin M., Sindi K. (2017). Specialised Sweetpotato Vine Multiplication in Lake Zone, Tanzania: What “Sticks” and What Changes?. Open Agric..

[bb0170] McNiven S., Gilligan D.O., Hotz C. (2016). Sustainability of impact: dimensions of decline and persistence in adopting a biofortified crop in Uganda. 3ie Impact Evaluation Report Series.

[bb0175] Ministério da Agricultura e Segurança Alimentar (2015). Annuário de Estatísticas Agrárias 2015.

[bb0180] Mozaffarian D., Rosenberg I., Uauy R. (2018). History of modern nutrition science—implications for current research, dietary guidelines, and food policy. BMJ.

[bb0185] Namanda S., Amour R., Gibson R.W. (2013). The Triple S method of producing sweet potato planting material for areas in Africa with long dry seasons. J. Crop Improv..

[bb0190] Ndirigwe J., Sindi K., Low J., Shumbusha D., Shingiro J.B., Nshimiyimana J.C., Hakizimana S., Angsten A., Low J., Nyongesa M., Quinn S., Parker M. (2015). Building a sustainable sweetpotato value chain: Experience from the Rwanda Sweetpotato Super Foods Project. Potato and Sweetpotato in Africa: Transforming the Value Chains for Food and Nutrition Security.

[bb0195] Okello J., Wanjohi L., Makokha P., Low J.W., Kwikiriza N. (2019). Status of Sweetpotato in Sub-Saharan Africa: September 2019. Sweetpotato for Profit and Health Initiative.

[bb0200] Phillips F., Hwang G., Limprayoon P. (2016). Inflection points and industry change: Was Andy Grove right after all?. J. Technol. Manage. Growing Econ..

[bb0205] Rajendran S., Kimenye L.N., McEwan M. (2017). Strategies for the development of the sweetpotato early generation seed sector in eastern and southern Africa. Open Agric..

[bb0210] Reddy V. (2002). History of the International Vitamin A Consultative Group 1975–2000. J. Nutr..

[bb0215] Rogers E.M. (2003). Diffusion of Innovations.

[bb0220] Rosen D.S., Haselow N.J., Sloan N.L. (1994). How to use the HKI food frequency method to assess community risk of vitamin A deficiency.

[bb0225] Ruel M.T., Levin C.E. (2000). Assessing the potential for food-based strategies to reduce vitamin A and iron deficiencies: A review of recent evidence.

[bb0230] Ruel M.T., Alderman H., Maternal, Child Nutrition Study, G (2013). Nutrition-sensitive interventions and programmes: how can they help to accelerate progress in improving maternal and child nutrition?. Lancet.

[bb8000] Ruel M.T., Quisumbing A.R., Balagamwala M., International Food Policy Research Institute (Ed.) (2017). Nutrition-Sensitive Agriculture: What Have We Learned and Where Do We Go From Here?.

[bb0235] Sartas M., Schut M., Stoian D., Velasco C., Campilan D., Thiele G., Leeuwis C. (2017). Scaling readiness: Accelerating the scaling of RTB interventions.

[bb0240] Stathers T., Mkumbira J., Low J.W., Tagwireyi J., Munyua H., Mbabu A., Mulongo G. (2015). Orange-fleshed Sweetpotato Investment Guide.

[bb0245] Stevens G.A., Bennett J.E., Hennocq Q., Lu Y., De-Regil L.M., Rogers L., Danaei G., Li G., White R.A., Flaxman S.R., Oehrle S.-P., Finucane M.M., Guerrero R., Bhutta Z.A., Then-Paulino A., Fawzi W., Black R.E., Ezzati M. (2015). Trends and mortality effects of vitamin A deficiency in children in 138 low-income and middle-income countries between 1991 and 2013: a pooled analysis of population-based surveys. Lancet Glob. Health.

[bb0250] The World Bank (2007). The World Development Report 2008: Agricuture for Development.

[bb0255] The World Bank (2014). Learning from World Bank Histiry: Agriculture and Food-Based Approaches for Addressing Malnutrition.

[bb0260] van Jaarsveld P., Faber M., Tanumihardjo S.A., Nestel P., Lombard C.J., Benade A.J. (2005). Beta-carotene-rich orange-fleshed sweet potato improves the vitamin A status of primary school children assessed with the modified-relative-dose-response test. Am. J. Clin. Nutr..

[bb0265] Walker T.S., Alwang J. (2015). Crop improvement, adoption, and impact of improved varieties of food crops in Sub-Saharan Afirca.

[bb0270] Woltering L., Fehlenberg K., Gerard B., Ubels J., Cooley L. (2019). Scaling - from "reaching many" to sustainable systems change at scale: A critical shift in mindset. Agric. Syst..

[bb0275] World Health Organization (2010). Nine steps for developing a scaling-up strategy.

[bb0280] World Health Organization (WHO) (2009). Global prevalence of vitamin A deficiency in populations at risk 1995–2005. WHO Global Database on Vitamin A Deficiency.

